# Supplementary material for: Attribution assignment for deep-generative sequence models enables interpretability analysis using positive-only data
Source: PLoS Comput Biol. 2026 Sep 28;22(9):e1014805. doi: 10.1371/journal.pcbi.1014805 (PMC13638040; doi:10.1371/journal.pcbi.1014805)
Supplement: Supplementary file 1 [file pcbi.1014805.s001.pdf]

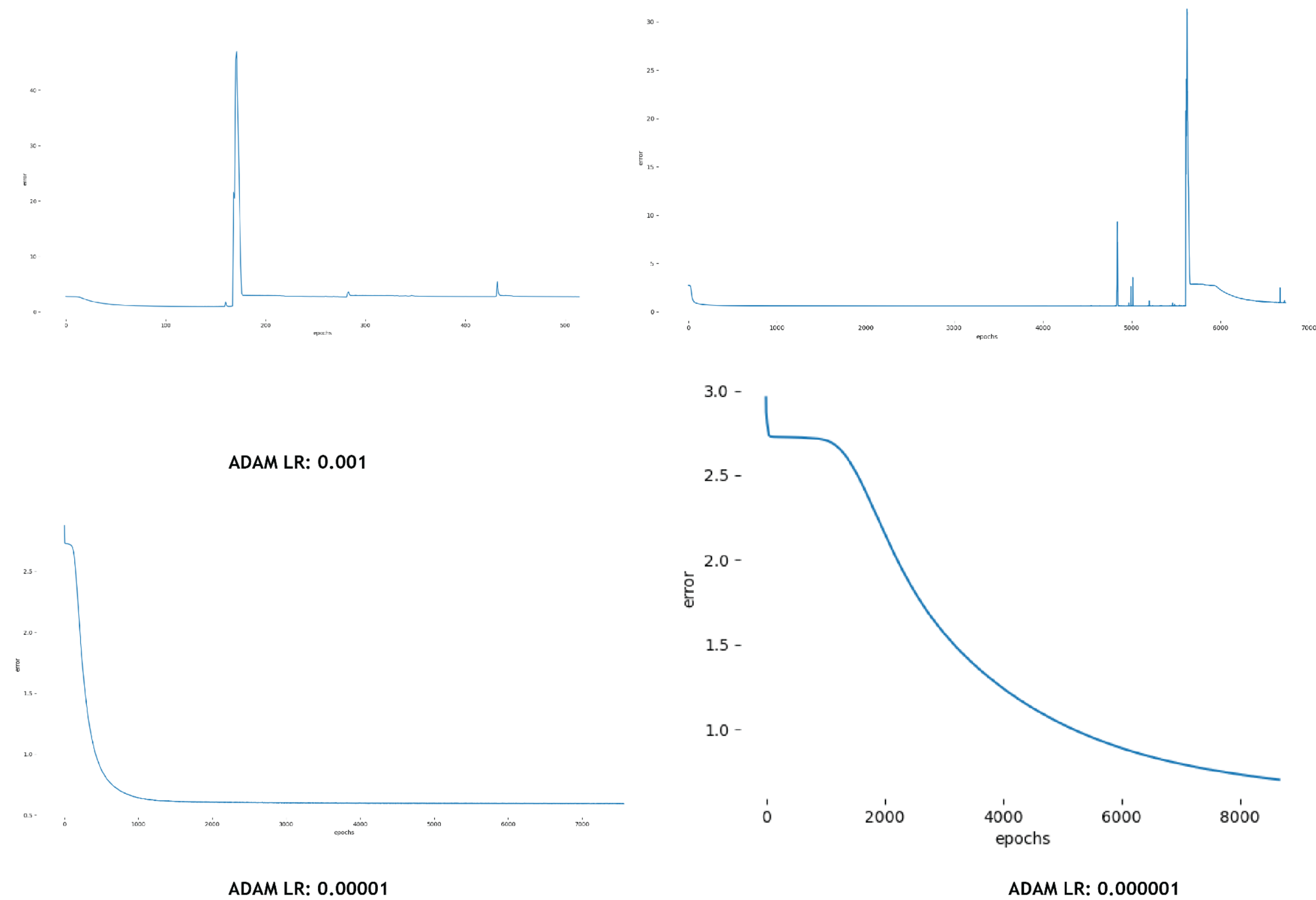

S1 Fig. A learning rate of 0.00001 is effective for training the LSTM on the synthetic dataset. Evaluating the influence of four different learning rates on the training stability on the synthetic dataset. The ADAM optimizer with a learning rate of 0.00001 showed a good trade-off between training speed and stability.
