# Supplementary material for: Attribution assignment for deep-generative sequence models enables interpretability analysis using positive-only data
Source: PLoS Comput Biol. 2026 Sep 28;22(9):e1014805. doi: 10.1371/journal.pcbi.1014805 (PMC13638040; doi:10.1371/journal.pcbi.1014805)
Supplement: Supplementary file 2 [file pcbi.1014805.s002.pdf]

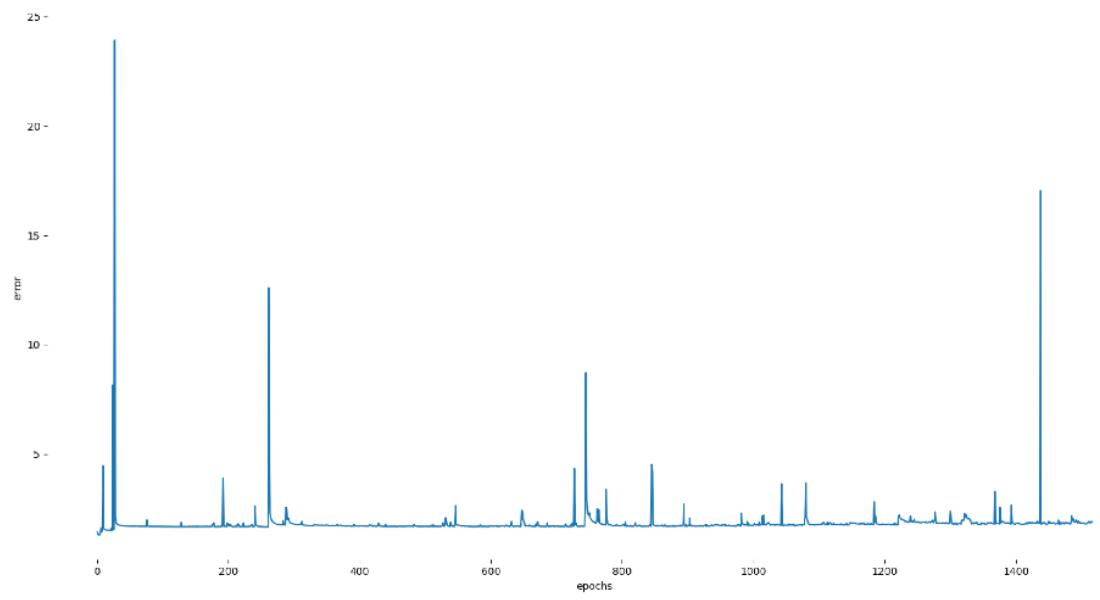

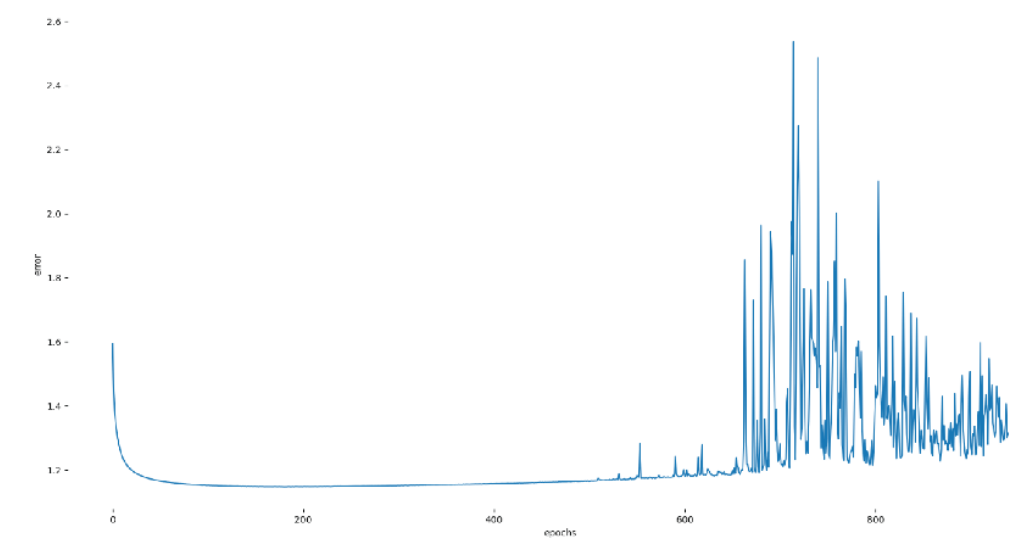

ADAM LR: 0.0001

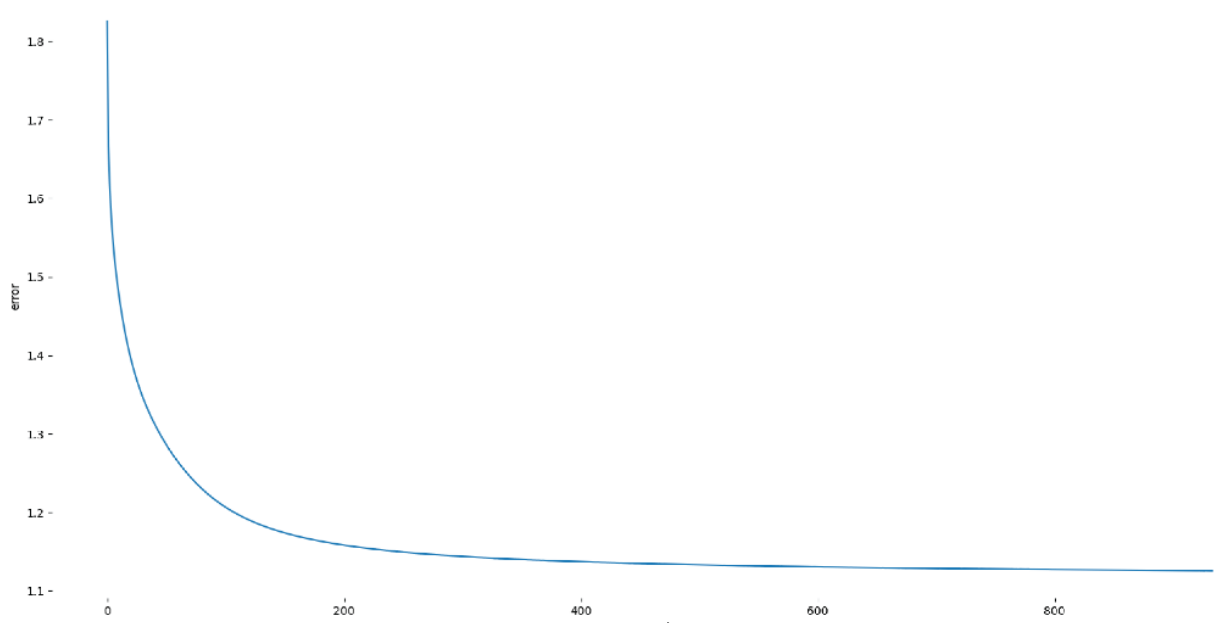

ADAM LR: 0.00001

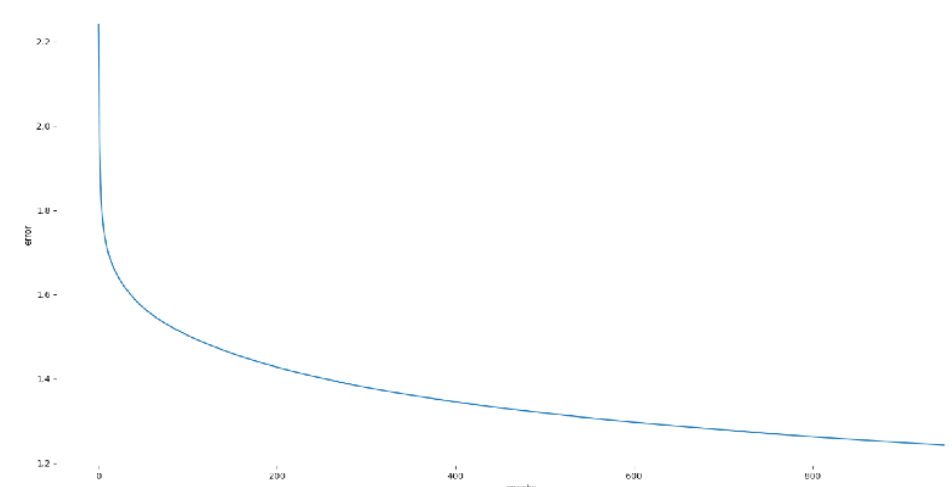

ADAM LR: 0.000001

S2 Fig. A learning rate of 0.00001 is effective for training the LSTM on the experimental dataset. Evaluating the influence of four different learning rates on the training stability on the experimental dataset. The ADAM optimizer with a learning rate of 0.00001 showed a good trade-off between training speed and stability.
