# Supplementary material for: Attribution assignment for deep-generative sequence models enables interpretability analysis using positive-only data
Source: PLoS Comput Biol. 2026 Sep 28;22(9):e1014805. doi: 10.1371/journal.pcbi.1014805 (PMC13638040; doi:10.1371/journal.pcbi.1014805)
Supplement: Supplementary file 3 [file pcbi.1014805.s003.pdf]

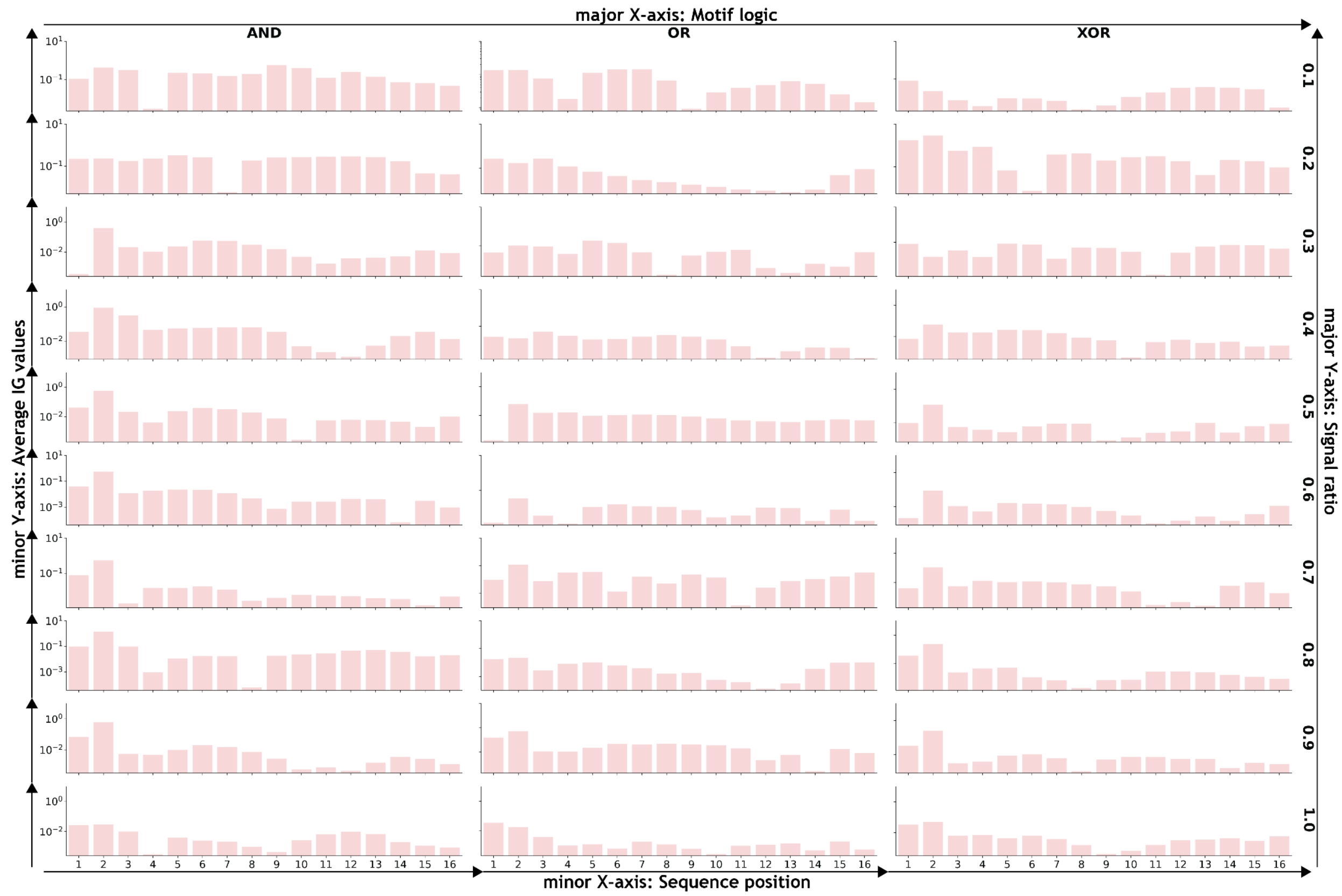

S3 Fig. The IG baseline is unsuitable for recovering relevant motifs at positions 2 and 4. This figure shows the false-negative rate of motif recovery using the IG baseline for datasets of uniformly random amino acid sequences with implanted motifs at positions 2 and 4. Each dataset contains 10 000 sequences of length sixteen. This panel is the IG-baseline counterpart of the GAMA experiment shown in Figure 4. In total, 30 datasets were generated, covering all combinations of motif logic (AND, OR, XOR) and signal ratios. The figure is a facet plot of bar charts. Each facet corresponds to a specific combination of motif logic (columns) and signal ratio (rows). Within each facet, bars show the IG-baseline values (as defined in the Methods) for all sequence positions on a logarithmic scale, with sequence position on the x-axis and IG-baseline magnitude on the y-axis. Across conditions, the IG baseline largely fails to highlight the true motif positions two and four, with only occasional recovery of position 2, indicating that this baseline is generally unsuitable for robust motif identification in this setting.
