# Supplementary material for: Attribution assignment for deep-generative sequence models enables interpretability analysis using positive-only data
Source: PLoS Comput Biol. 2026 Sep 28;22(9):e1014805. doi: 10.1371/journal.pcbi.1014805 (PMC13638040; doi:10.1371/journal.pcbi.1014805)
Supplement: Supplementary file 10 [file pcbi.1014805.s010.pdf]

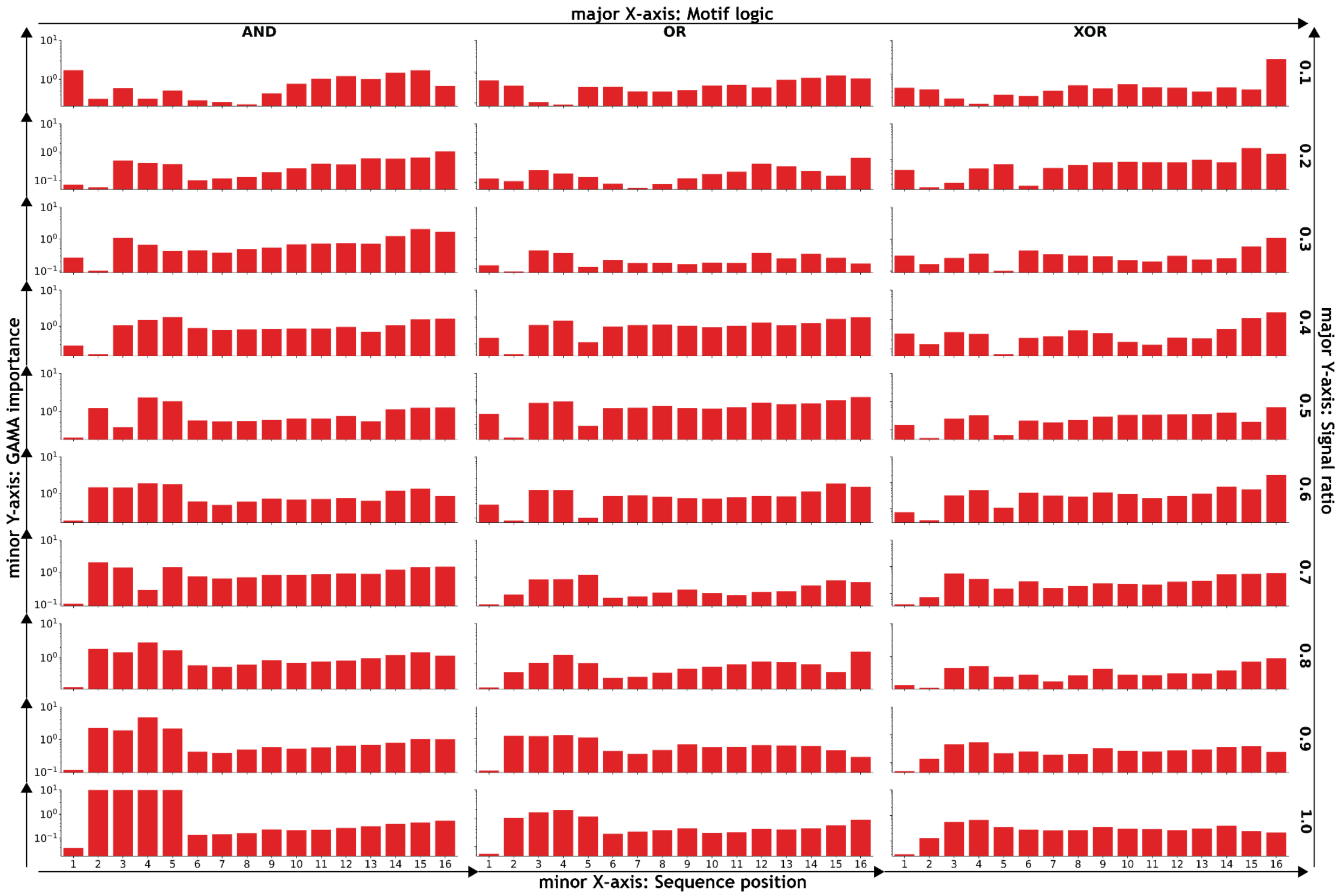

S10 Fig. GAMA attribution magnitude decreases with higher noise ratios at positions 2, 3, 4, and 5. This figure shows the GAMA importance values for datasets of uniformly random amino acid sequences with implanted motifs at positions 2, 3, 4 and 5. Each dataset contains 10 000 sequences of length sixteen. In total, 30 datasets were generated, covering all combinations of motif logic (AND, OR, XOR) and signal ratios. The figure is a facet plot of bar charts. Each facet corresponds to a specific combination of motif logic (columns) and signal ratio (rows). Within each facet, bars show GAMA importance values (as defined in the Methods) for all sequence positions on a logarithmic scale, with sequence position on the x-axis and GAMA magnitude on the y-axis. Across conditions, GAMA places greater importance on the true motif positions 2, 3, 4 and 5, particularly at high signal ratios. As the proportion of noise sequences increases, GAMA values at the motif positions decrease, while values at non-motif positions remain relatively constant, indicating that GAMA is sensitive to the implanted motif signal and degrades gracefully with increasing noise. Supplementary figure 11 depicts the same visualization for the IG baseline.
