# Supplementary material for: Attribution assignment for deep-generative sequence models enables interpretability analysis using positive-only data
Source: PLoS Comput Biol. 2026 Sep 28;22(9):e1014805. doi: 10.1371/journal.pcbi.1014805 (PMC13638040; doi:10.1371/journal.pcbi.1014805)
Supplement: Supplementary file 12 [file pcbi.1014805.s012.pdf]

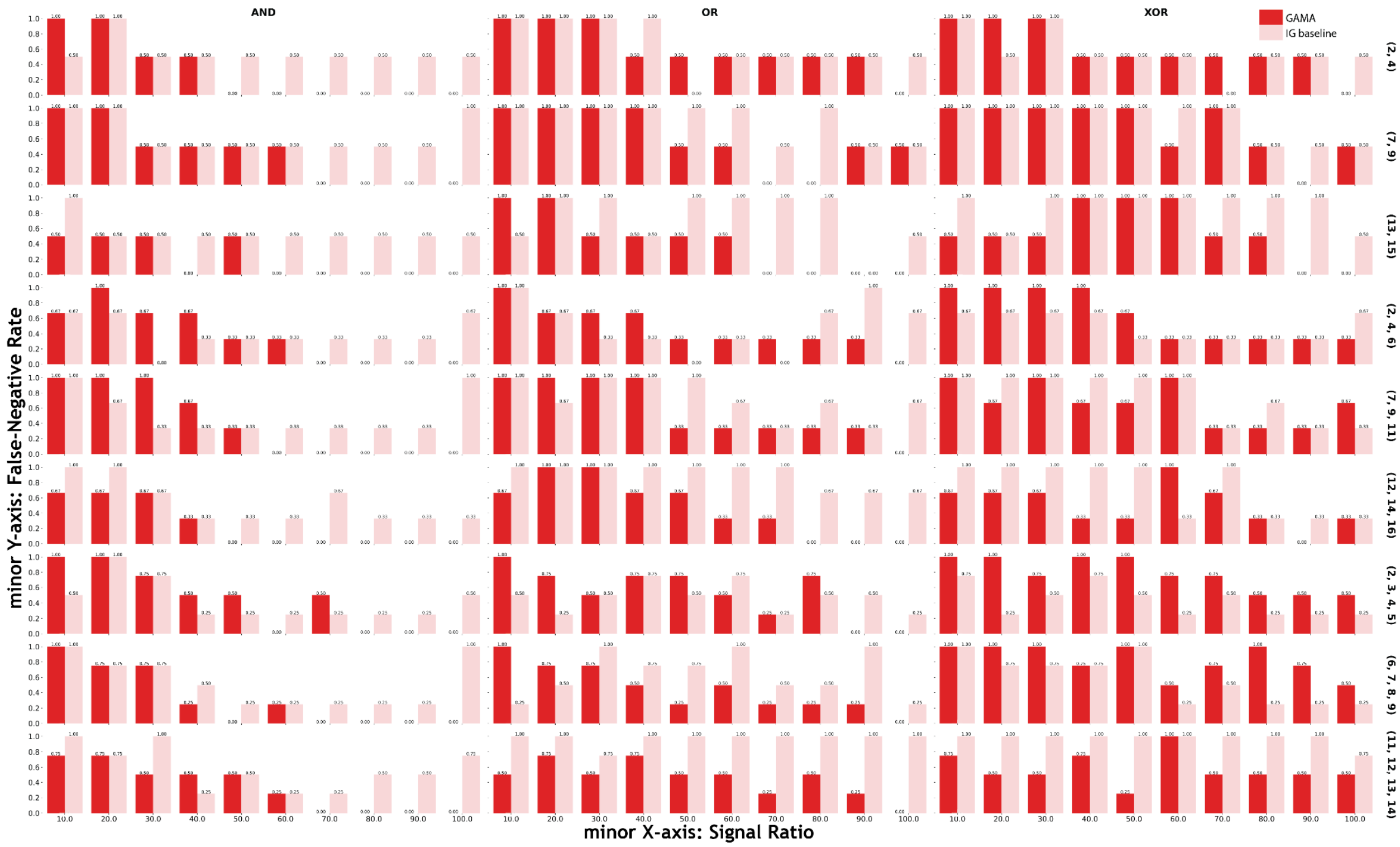

S12 Fig. Enhanced retrieval efficiency for motifs positioned at the end of sequences, measured with false-negative-rate. Retrieval performance of GAMA across motif positions. This figure summarizes motif-recovery performance for motifs positions. The facets are organized by motif position (rows) and motif logic (columns: AND, OR, XOR). Within each facet, bar heights indicate the false-negative rate (y-axis) for GAMA (red) and IG baseline (light red) at different signal ratios (x-axis). Each bar corresponds to one synthetic dataset (experimental condition), covering all 270 combinations of signal ratio, motif position, motif length, and motif logic. The second bar (light red) shows the corresponding false-negative rates for the IG baseline. Together, the bars reveal that GAMA achieves lower false-negative rates than the IG baseline for AND and OR logic experiments with less than 50% signal ratio.
