# Supplementary material for: Attribution assignment for deep-generative sequence models enables interpretability analysis using positive-only data
Source: PLoS Comput Biol. 2026 Sep 28;22(9):e1014805. doi: 10.1371/journal.pcbi.1014805 (PMC13638040; doi:10.1371/journal.pcbi.1014805)
Supplement: Supplementary file 13 [file pcbi.1014805.s013.pdf]

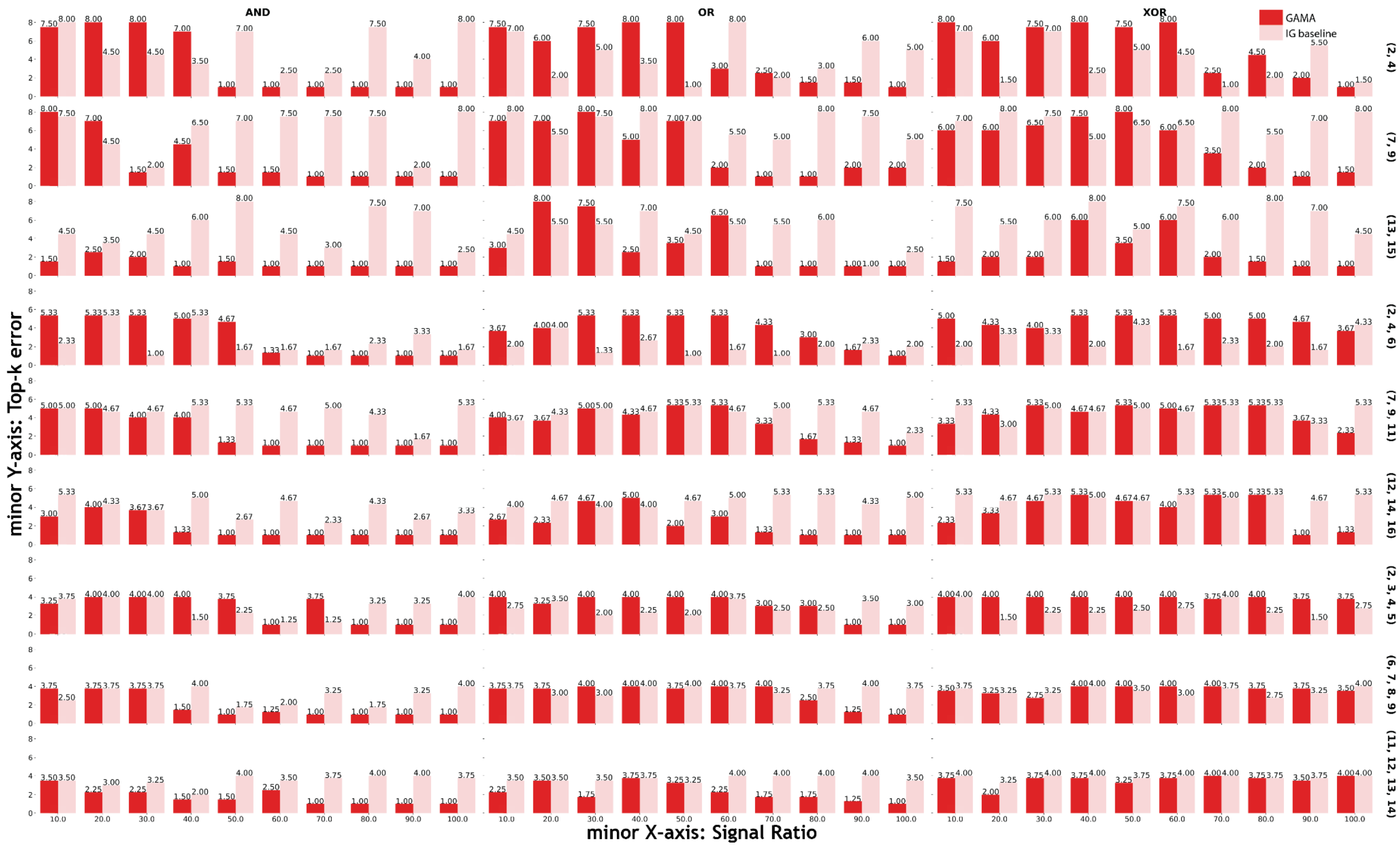


**S13 Fig. Enhanced retrieval efficiency for motifs positioned at the end of sequences, measured with top-k metric.** Retrieval performance of GAMA across motif positions. This figure summarizes motif-recovery performance for motifs positions. The facets are organized by motif position (rows) and motif logic (columns: AND, OR, XOR). Within each facet, bar heights indicate the top-k metric (y-axis) for GAMA (red) and IG baseline (light red) at different signal ratios (x-axis). Each bar corresponds to one synthetic dataset (experimental condition), covering all 270 combinations of signal ratio, motif position, motif length, and motif logic. The second bar (light red) shows the corresponding top-k values for the IG baseline. Together, the bars reveal that GAMA achieves better top-k values than the IG baseline for AND and OR logic that <50% signal ratio. The top-k error metric is defined as the fraction of true motif positions that lie outside the set of the top-k ranked positions (lower values indicate better retrieval performance). The top-k error metric is more fine-grained and reveals the magnitude of GAMA's outperformance over the IG baseline. It also reveals outperformance for positions that were not apparent with the false-negative rate (see Supplementary figure 12).
