# Supplementary material for: Attribution assignment for deep-generative sequence models enables interpretability analysis using positive-only data
Source: PLoS Comput Biol. 2026 Sep 28;22(9):e1014805. doi: 10.1371/journal.pcbi.1014805 (PMC13638040; doi:10.1371/journal.pcbi.1014805)
Supplement: Supplementary file 14 [file pcbi.1014805.s014.pdf]

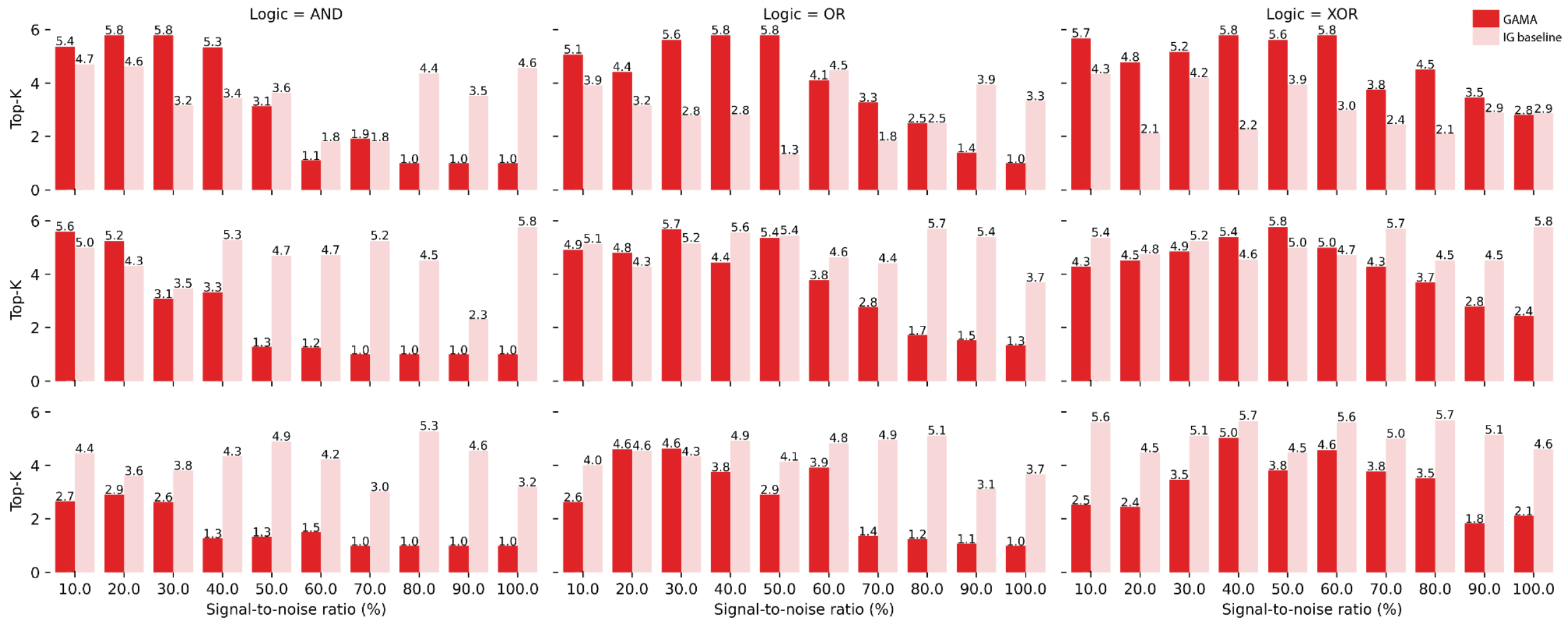

S14 Fig. Enhanced retrieval efficiency for motifs positioned at the end of sequences. The bar charts present the top-k position metric, which is defined as the count of amino acid positions from the top of the list, as determined by GAMA or the IG baseline, until the entire motif is recovered, with smaller values indicating better performance. The secondary y-axis depicts the top-k metric, while the secondary x-axis represents the signal ratio. The primary x-axis illustrates the results for three motif-logic variants: AND, OR, and XOR. The primary y-axis compares motifs located at the front, middle, and end of sequences, with values obtained by averaging different motif lengths. The figure indicates that motif recovery rates are superior when motifs are positioned at the end of the sequence.
