# Supplementary material for: Attribution assignment for deep-generative sequence models enables interpretability analysis using positive-only data
Source: PLoS Comput Biol. 2026 Sep 28;22(9):e1014805. doi: 10.1371/journal.pcbi.1014805 (PMC13638040; doi:10.1371/journal.pcbi.1014805)
Supplement: Supplementary file 15 [file pcbi.1014805.s015.pdf]

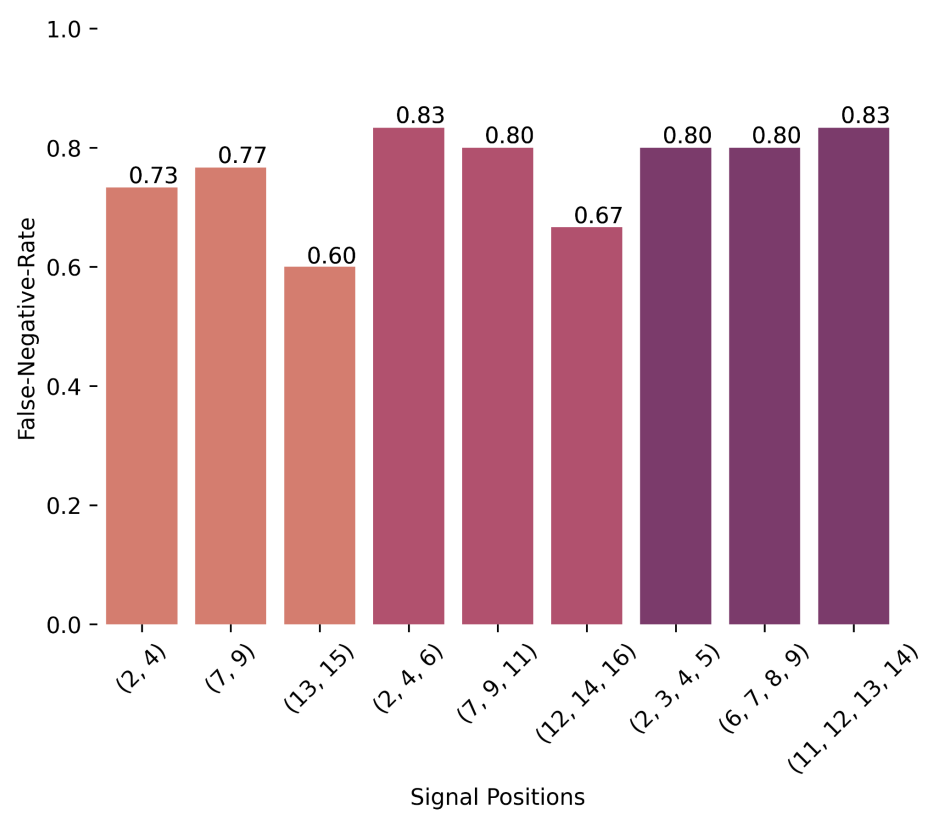

S15 Fig. Retrieval performance improves for shorter motifs. The y-axis indicates the false-negative-rate and the x-axis indicates the motif type and position. The three 2-position motifs have lower false-negative-rates than the longer (3-and-4-position)-motifs.
