# Supplementary material for: Attribution assignment for deep-generative sequence models enables interpretability analysis using positive-only data
Source: PLoS Comput Biol. 2026 Sep 28;22(9):e1014805. doi: 10.1371/journal.pcbi.1014805 (PMC13638040; doi:10.1371/journal.pcbi.1014805)
Supplement: Supplementary file 16 [file pcbi.1014805.s016.pdf]

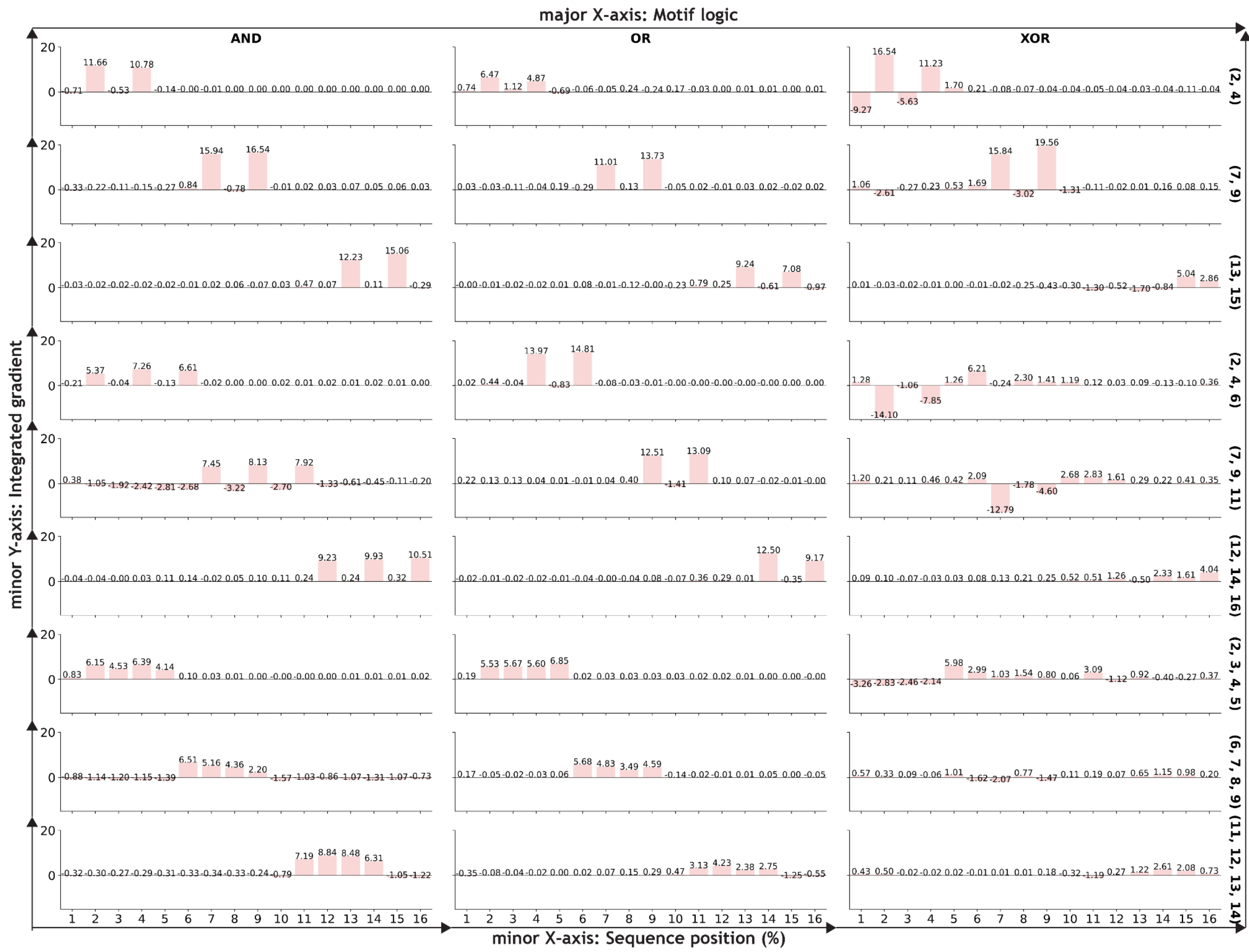

S16 Fig. **Integrated gradients on supervised LSTM binary classifiers reliably recover AND and OR motifs but fail on XOR logic.** Integrated gradients (IG) baseline on supervised synthetic motif classification, contrasting with our unsupervised GAMA setup. Here, we train a single-layer LSTM (hidden size 1024, followed by a fully connected layer and logistic output) on the same synthetic sequences, but now with explicit labels: signal sequences as positive and noise sequences as negative (10 000 each). In this supervised setting, the task is trivial: signal and noise are perfectly labeled, classes are balanced, and noise sequences are uniformly random, so the presence or absence of the planted motif almost perfectly separates the two classes. Under these conditions, classification error quickly converges to zero. We then compute IG on all 20 000 training sequences and average attributions across sequences. Each panel shows the average IG for a given motif-logic condition (major x-axis: AND, OR, XOR) and motif location (major y-axis; motif positions indicated). Within panels, bars show the mean IG across sequence positions (x-axis), with bar height and overlaid numeric labels indicating the mean IG (y-axis). Under these supervised conditions, IG perfectly recovers motif positions for AND logic and almost perfectly for OR logic, failing only to highlight one position in the (7, 9, 11) and (12, 14, 16) motifs. In contrast, most XOR configurations still yield poor motif recovery with large negative attributions at relevant positions, underscoring that even with ideal supervision and labels, IG struggles to faithfully represent XOR-like interactions compared to the unsupervised GAMA results.
