# Supplementary material for: Attribution assignment for deep-generative sequence models enables interpretability analysis using positive-only data
Source: PLoS Comput Biol. 2026 Sep 28;22(9):e1014805. doi: 10.1371/journal.pcbi.1014805 (PMC13638040; doi:10.1371/journal.pcbi.1014805)
Supplement: Supplementary file 17 [file pcbi.1014805.s017.pdf]

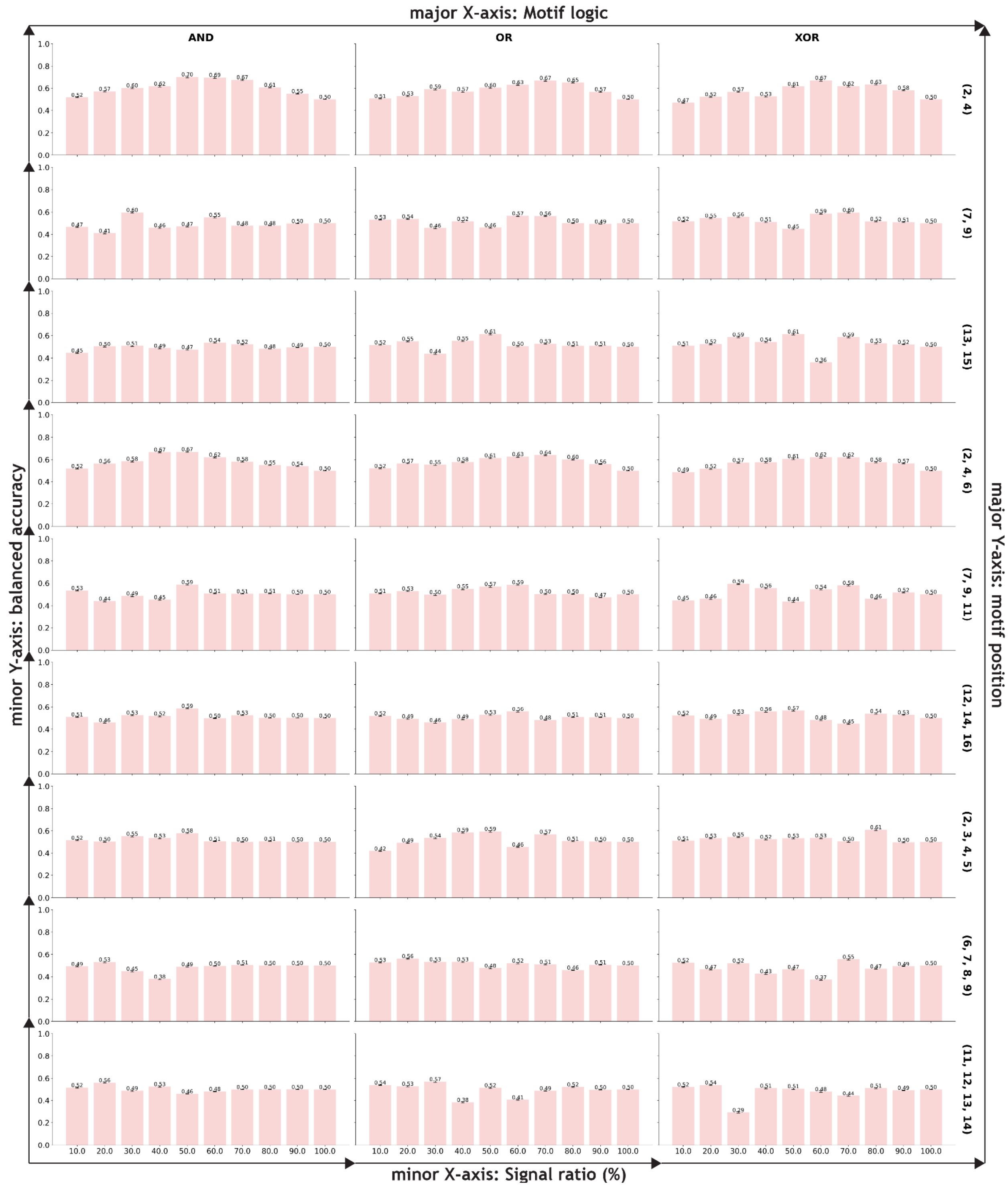

S17 Fig. **Positive-Only IsolationForest Fails to Reliably Classify Synthetic Motif Sequences.** IsolationForest baseline on the synthetic motif dataset used for GAMA. We trained a positive-only IsolationForest classifier (scikit-learn) on 10 000 signal sequences plus a variable number of noise sequences, generated with random seed = 0, depending on the signal ratio. Sequences were one-hot encoded, and the IsolationForest contamination parameter was set to match the true signal ratio for each condition. We used n_estimators=1000 and max_samples=1024, with all other hyperparameters at their scikit-learn defaults. This figure provides an indirect baseline: IsolationForest is a sequence-level positive-only classifier, whereas GAMA evaluates the retrieval of specific motif features within sequences; thus, their performance metrics are not directly comparable. Performance was

assessed on 1 000 held-out signal sequences and 1 000 held-out noise sequences (generated with seed = 1), using balanced accuracy to ensure results were comparable across signal ratios. Facets are organized by motif position (rows) and motif logic (columns: AND, OR, XOR). Within each facet, bars show balanced accuracy across different signal ratios (x-axis), with bar height and overlaid numeric labels indicating balanced accuracy (y-axis). Error bars denote 95% confidence intervals computed from Wilson score intervals for TPR and TNR, with balanced-accuracy variability obtained via Newcombe's method. For longer motifs, balanced accuracy is generally near chance (ca. 0.5), while shorter motifs occasionally yield moderate performance. Overall, the IsolationForest classifier performs poorly on this task.
