# Supplementary material for: Attribution assignment for deep-generative sequence models enables interpretability analysis using positive-only data
Source: PLoS Comput Biol. 2026 Sep 28;22(9):e1014805. doi: 10.1371/journal.pcbi.1014805 (PMC13638040; doi:10.1371/journal.pcbi.1014805)
Supplement: Supplementary file 19 [file pcbi.1014805.s019.pdf]

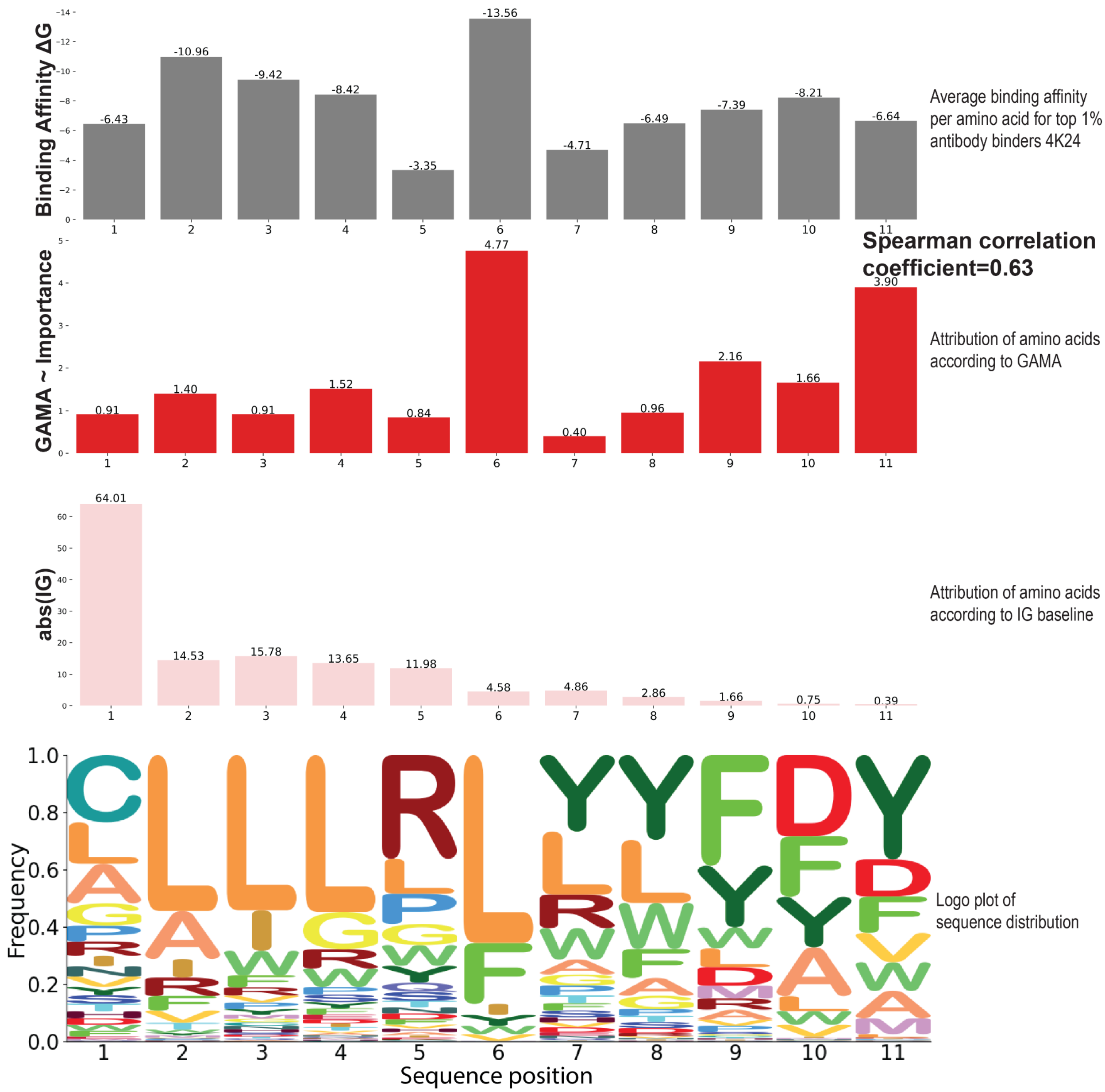

S19 Fig. Absolut! estimated amino acid-level binding affinities correlate with GAMA derived importance scores for the 4K24 antigen. Mean amino acid binding affinities and of 2173 CDRH3 sequences of the top 1% binders to the 4K24 antibody and attribution assignment according to GAMA have a Spearman correlation of 0.63. The IG baseline fails to identify important features.
