# Supplementary material for: Attribution assignment for deep-generative sequence models enables interpretability analysis using positive-only data
Source: PLoS Comput Biol. 2026 Sep 28;22(9):e1014805. doi: 10.1371/journal.pcbi.1014805 (PMC13638040; doi:10.1371/journal.pcbi.1014805)
Supplement: Supplementary file 21 [file pcbi.1014805.s021.pdf]

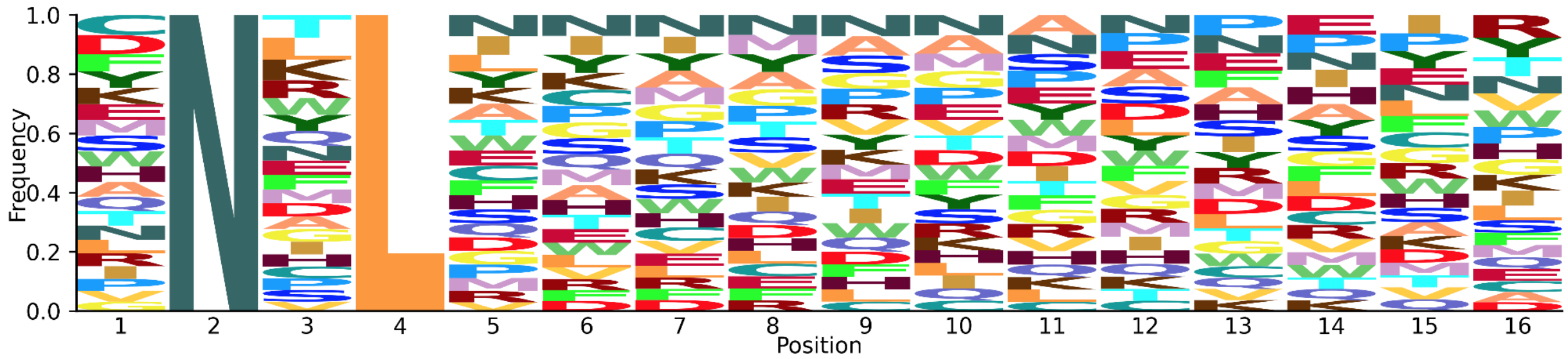

S21 Fig. Generative LSTM models trained on the synthetic datasets produce representative samples of the training distribution. Logoplot of the frequency distribution of 10 000 samples, sampled with a temperature parameter of 0.5, from a LSTM trained on a synthetic dataset with implanted motifs at positions two and four, “AND” logic interaction, and 100% signal ratio.
