# Supplementary material for: Attribution assignment for deep-generative sequence models enables interpretability analysis using positive-only data
Source: PLoS Comput Biol. 2026 Sep 28;22(9):e1014805. doi: 10.1371/journal.pcbi.1014805 (PMC13638040; doi:10.1371/journal.pcbi.1014805)
Supplement: Supplementary file 22 [file pcbi.1014805.s022.pdf]

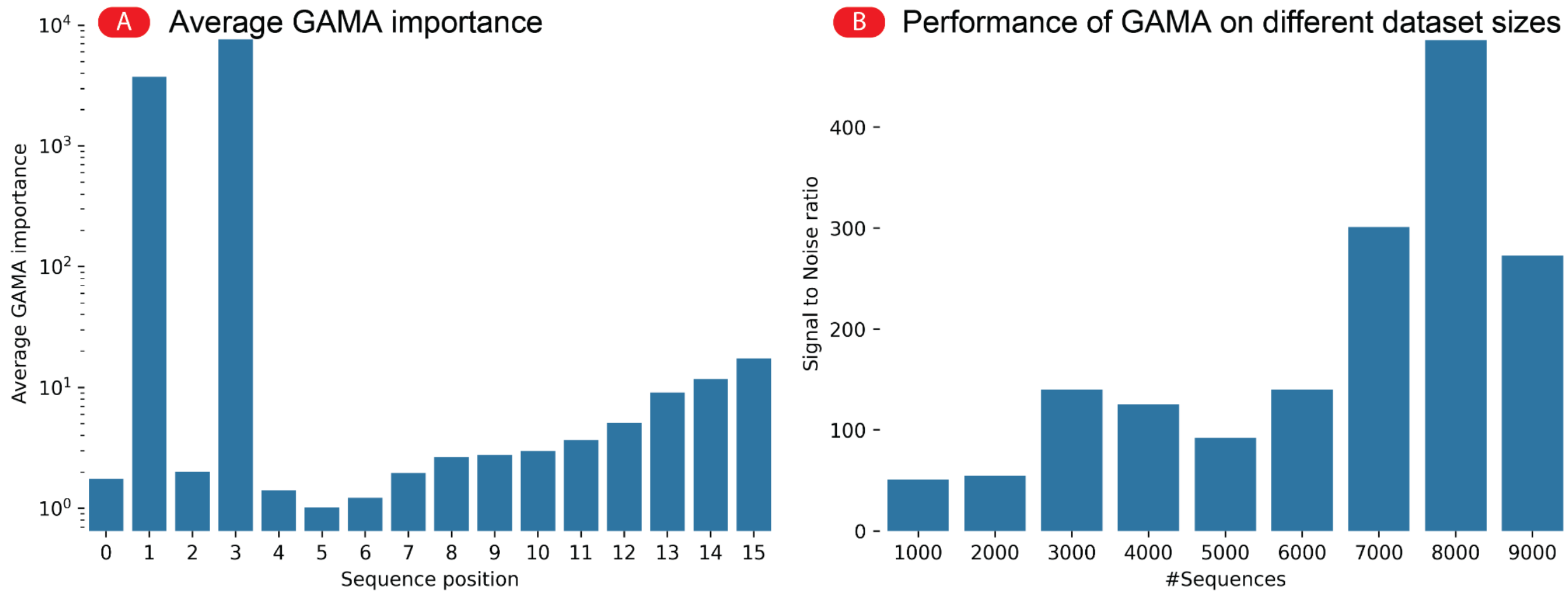

S22 Fig. A larger number of sequences results in stronger GAMA signals. We computed GAMA for nine synthetic datasets with a size between 1000 and 9000 sequences. All datasets had a signal implanted at positions two and four, motif positions interact with "AND" logic, and had no noise sequences. For all nine datasets, GAMA identifies the motif positions correctly. (A) This panel shows the average GAMA importance on a log scale, for each sequence position, over all nine datasets. A strong differentiation for the implanted motif positions can be seen at two and four.

(B) In this panel we investigated the ratio of GAMA importance values at positions where the motif was implanted (two and four) versus the GAMA at non-motif positions. Even datasets with as little as 1000 sequences, lead to signal ratios far larger than one.
