# Supplementary material for: Attribution assignment for deep-generative sequence models enables interpretability analysis using positive-only data
Source: PLoS Comput Biol. 2026 Sep 28;22(9):e1014805. doi: 10.1371/journal.pcbi.1014805 (PMC13638040; doi:10.1371/journal.pcbi.1014805)
Supplement: Supplementary file 23 [file pcbi.1014805.s023.pdf]

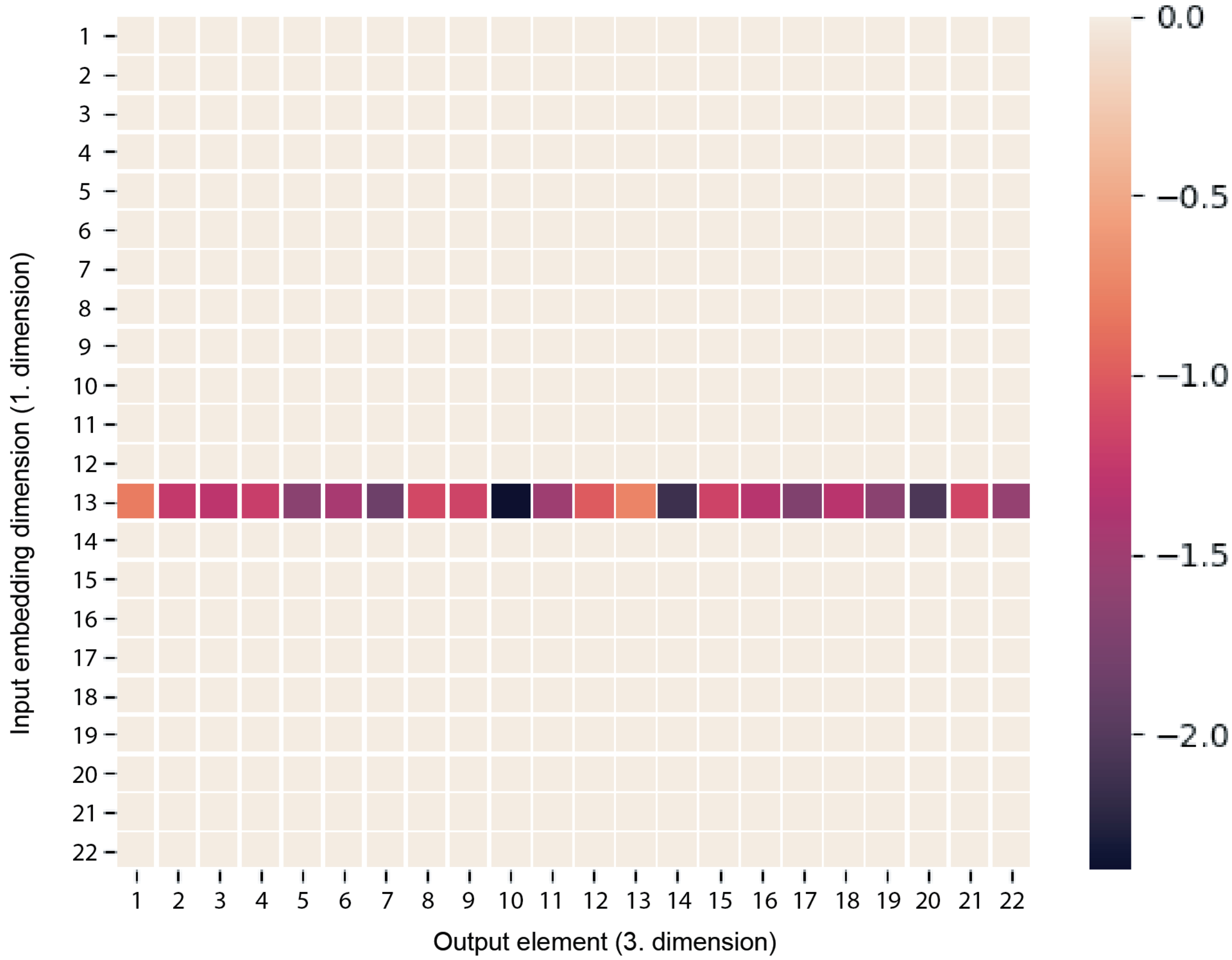

S23 Fig. IG values computed for the 22 output dimensions are similar. The heatmap values show IGs for a single sequence w.r.t. to one input token and computed for the next output token. The 22 encoding dimensions are displayed on the y-axis. The output dimension for which the gradient is computed, is displayed on the x-axis. The activation pattern is typical for all possible combinations of input-output combinations of the LSTM. The sequence displayed here had the input token 12 encoded as input. We can see that only IGs of row 12 are activated. The four dimensional IG tensor is reduced to a 2d tensor by selecting row 12 and averaging over it.
