# Supplementary material for: Attribution assignment for deep-generative sequence models enables interpretability analysis using positive-only data
Source: PLoS Comput Biol. 2026 Sep 28;22(9):e1014805. doi: 10.1371/journal.pcbi.1014805 (PMC13638040; doi:10.1371/journal.pcbi.1014805)
Supplement: Supplementary file 24 [file pcbi.1014805.s024.pdf]

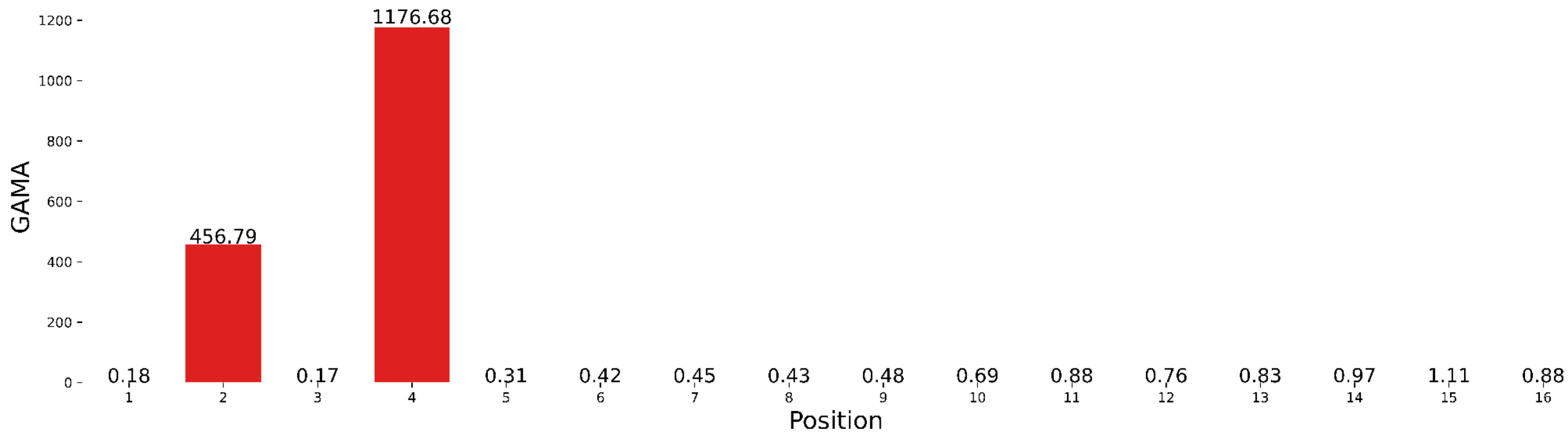

S24 Fig. The experiments on synthetic data show very similar results on different seeds. The barplot shows GAMA values for the synthetic experiment with signal positions at **two** and **four**, "AND" logic, and a signal ratio of one, repeated with a seed=1. In comparison to Figure 4, lower left corner which is the same experiment but was computed with seed=0.
